# Significance of Fib4 index as an indicator of alcoholic hepatotoxicity in health examinations among Japanese male workers: a cross-sectional and retrospectively longitudinal study

**DOI:** 10.1186/s40001-022-00976-6

**Published:** 2023-01-18

**Authors:** Hideki Shinoda, Yuya Watanabe, Kota Fukai, Kayoko Kasuya, Yuko Furuya, Shoko Nakazawa, Toru Honda, Takeshi Hayashi, Toru Nakagawa, Masayuki Tatemichi, Masaaki Korenaga

**Affiliations:** 1 Hitachi General Hospital, Hitachi, Japan; 2 Hitachi Health Care Center, Hitachi, Japan; 3 Department of Preventive Medicine, Tokai University School of Medicine, Isehara, Japan; 4 Hepatitis Information Centre, Research Centre for Hepatitis and Immunology, National Centre for Global Health and Medicine, Ichikawa, Japan; 5 Present Address: Occupational Hygiene and Promotion Center, Hitachi, Ltd, Tokyo, Japan

**Keywords:** Fib4 index (Fib4), Alcohol consumption, Gamma-glutamyl transferase (GGT), Alcoholic liver disease (ALD), Metabolic-associated fatty liver disease (NAFLD)

## Abstract

**Background:**

Fib4 index (Fib4) is clinically used as a noninvasive marker of liver fibrosis. In this study, we aimed to preliminarily investigate whether Fib4 can be used to detect individuals who need assessment for alcoholic liver disease (ALD) in the general population by clarifying the detailed association of Fib4 with alcohol consumption and gamma-glutamyl transferase (GGT) among male workers.

**Methods:**

We analyzed data sets on the comprehensive medical examinations of male workers as cross-sectional and retrospectively longitudinal studies. We enrolled 10 782 males (mean age: 52.2 ± 10.2 years) in FY2019 and 7845 males (mean follow-up: 12.6 ± 6.7 years) who could be consecutively followed up for 20 years from FY2000 to FY2019. Data were evaluated using logistic regression and COX proportional analysis.

**Results:**

In the cross-sectional setting, the rate of Fib4 ≥ 2.67 in heavy drinkers (≥ 40 g of ethanol/day) was increased dose dependently in those over 65 years old, and that of body mass index ≥ 30 kg/m^2^ was increased in those over 60 years old, but not in those with fatty liver. The odds ratio (OR) (95% confidence interval [CI]) for heavy drinking was 4.30 (95% CI = 1.90–9.72), and GGT ≥ 200 IU/L was considerably high (OR = 29.05 [95% CI = 17.03–49.56]). In the longitudinal setting, heavy drinkers and those with GGT ≥ 200 IU/L at 10 years after the baseline showed an increased risk for Fib4 ≥ 2.67 (hazard ratio = 2.17 [95% CI = 1.58–2.98] and 7.65 [95% CI 5.26–11.12], respectively).

**Conclusions:**

The development of Fib4 ≥ 2.67 after 10 years was associated with heavy alcohol drinking and GGT level ≥ 200 IU/L. Therefore, Fib4 combined with GGT could indicate high risk of ALD. However, clinical examinations and course observations are essentially needed.

**Supplementary Information:**

The online version contains supplementary material available at 10.1186/s40001-022-00976-6.

## Background

The Fib4 index (Fib4) proposed by Sterling et al. has been developed as a simple index of liver fibrosis that can be calculated by adding platelets to age, aspartate aminotransferase (AST), and alanine aminotransferase (ALT) [1]. Recently, nonalcoholic fatty liver disease (NAFLD) has become a major concern in not only liver disease but also metabolic syndrome and cardiovascular events [2]. NAFLD is divided into nonalcoholic fatty liver (NAFL) and nonalcoholic steatohepatitis (NASH) [3]. Given that patients with NASH are at risk of developing liver fibrosis to hepatocellular carcinoma (HCC), Fib4 is recommended as a clinical marker for easily assessing the degree of progression of liver fibrosis in patients with NAFLD [4].

In the clinical setting of NAFLD management, Fib4 is popularly used by hepatologists [5], and several studies applied Fib4 in the general population [6, 7]. Regarding workers’ health examinations in Japan, blood count tests only collect data on red blood cells in order to assess anemia. In contrast, platelet (and white blood cells) counts are typically estimated automatically in blood count tests, excluding information that could be clinically important. Consequently, the current study was aimed to capitalize on this platelet data among Japanese workers. Originally, Fib4 was developed as a marker of fibrosis caused by hepatitis C [1] and was subsequently associated with viral hepatitis. However, liver fibrosis is not simply caused by viral hepatitis or NASH. Fib4 changes can be caused by other liver disorders, including alcoholic liver disease (ALD), considering that 27% of deaths caused by cirrhosis or chronic liver disease were reported to be linked to ALD [6]. Patients with ALD exhibit increased AST/ALT ratio, markedly increased gamma-glutamyl transferase (GGT), decreased cholinesterase, increased fibrosis marker, and decreased PT levels [8].

Symptoms are unlikely to appear unless ALD progresses [8]. Thus, detecting individuals who are at an early stage of ALD is necessary. ALD occurs in individuals with long-term excessive drinking, that is, drinking beverages containing ≥ 60 g of ethanol per day for ≥ 5 years [9]. However, Corrao et al. reported that people consuming 25 g of ethanol per day have a significantly increased risk for liver cirrhosis compared with abstainers [10]. Given that heavy drinkers frequently claim less alcohol consumption [11, 12], objective evaluation by hearing from a third party (family, friends, work colleagues, etc.) is also required. However, hearing from the family is usually difficult during medical examination, the GGT level is used as a popular marker of alcohol consumption in some populations [13, 14]

The pathological progression of NASH and ALD is similarly thought to be mediated by reactive oxygen species [15]. However, Fib4 as an effect marker of liver damage by alcoholic consumption among the general population remains unconfirmed. Therefore, to preliminarily investigate the significance of Fib4 in the general population in detecting patients that need assessment for ALD, we clarified the detailed association of Fib4 with alcohol consumption and GGT by using cross-sectional and longitudinal methods with 20 years of follow-up period.

## Methods

### Subjects

This study was conducted at a health center affiliated to a group of large-scale companies. Employees and their spouses from approximately 30 affiliated companies (30,000 employees) freely selected the timing and health center for their comprehensive health examinations. Details were described previously [16–20]. In the present study, we analyzed two data sets of comprehensive medical examinations as cross-sectional and longitudinal studies in the following years, from fiscal year (FY), which starts from April in Japan, 2000 to FY2019. First, we obtained the most current data of 15,792 examinees at FY2019, consisting of 13,700 males and 2092 females (mean age ± standard deviation [SD] = 53.0 ± 10.0). Second, 16,408 examiners (males = 13,701 and females = 2707; mean age = 47.8 ± 9.2 years) at FY2000 were obtained as the baseline and were followed up yearly until FY2019.

**Fig. 1:**
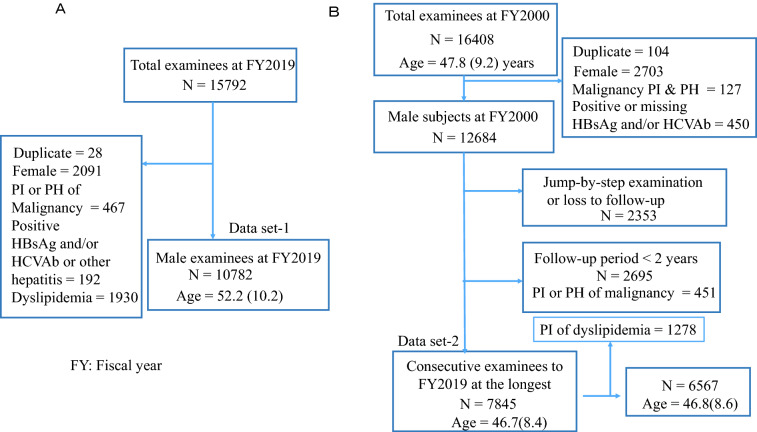
Data set diagram. **A** Cross-sectional data set. **B** Follow-up data set

Due to the small number of females available for follow-up and their lower drinking habits, only men were enrolled in this study. To identify liver dysfunction-associated factors, such as ALT and AST, among 13,700 male examinees at FY2019, we established data set-1 (mean age ± SD = 53.1 ± 10.3), which included 12,918 examinees. Those with a present or past history of malignancy, hepatitis, dyslipidemia, positive HBsAg, or positive HCVAb were excluded. We excluded examinees with a present or past history of malignancy because some anticancer drugs may affect the platelet count. We also excluded those with a present illness of dyslipidemia because we preliminarily found possible associations between HDL or LDL and Fib4. We defined dyslipidemia as a person who is taking medication for dyslipidemia. In the longitudinal analysis, out of 13,459 male examinees at FY2000, 7845 were consecutively examined until FY2019, constituting the data set-2 (mean age ± SD = 46.7 ± 8.4), which had a mean follow-up period of 12.1 ± 6.0 (SD) years. Figure 1 presents the diagrams of the data sets.

Information on the present and/or past history of illness, smoking, and alcohol drinking was obtained using a health questionnaire. Total amount of alcohol consumption was calculated using data on weekly frequency and daily amount of consumption of alcoholic beverages. Then alcohol drinking was categorized to never-drinker, < 20, 20–40, > 40 g-ethanol/day, equivalent to < 1, 1 to 2, > 2 *go* of Japanese sake per day [21].

This study was approved by the Institutional Review Board for Clinical Research in Tokai University (20R369) and the Hitachi Review Board (2021–16).

### Statistical analysis

#### Risk factors of Fib4 ≥ 2.67 in a cross-sectional setting

Fib4 index was calculated using the following formula [1]:

$${\text{Fib4 index}} = [{\text{Age}} \, ({\text{years}}) \times {\text{AST}}(U/L)]/[{\text{platelet}} \, (109/L) \times \surd {\text{ALT(U/L}}].$$

A Fib4 index of < 1.3, 1.3–2.67, or ≥ 2.67 was considered as a low, moderate, or high risk for fibrosis, respectively [22, 23]. The rate of patients with Fib4 ≥ 1.3 or ≥ 2.67 was calculated by age group.

In the data set of FY 2019, the odds ratios (ORs) and 95% confidence interval (CI) of the risk for Fib4 ≥ 2.67 (high risk) were calculated using the logistic model. The selected variables were liver dysfunction-related factors, such as body mass index (BMI), fatty liver detected by ultrasonography, abdominal condition, alcohol drinking, and GGT. According to a preliminary univariate analysis, HDL and LDL showed a significant association, thereby included as variables. Smoking history was also considered as a variable because of its association with fibrosis [2].

#### Risk factors of Fib4 ≥ 2.67 in a retrospective cohort setting

To identify the risk factors for the outcome of Fib4 ≥ 2.67 even once from FY2000 to FY2019 in the data set-2, we calculated the hazard ratio (HR) and 95% CI by using the COX proportional hazard model. In particular, we calculated the HR and 95% CI of the variables age, BMI, alcohol drinking history, GGT, HDL, and LDL as covariates, as examined in previous studies [5]. Finally, these variables were entered into the COX model. These variables from the data obtained in FY2010 and FY2019 were also entered. All statistical data were analyzed IBM-SPSS version 28.

## Results

**Fig. 2:**
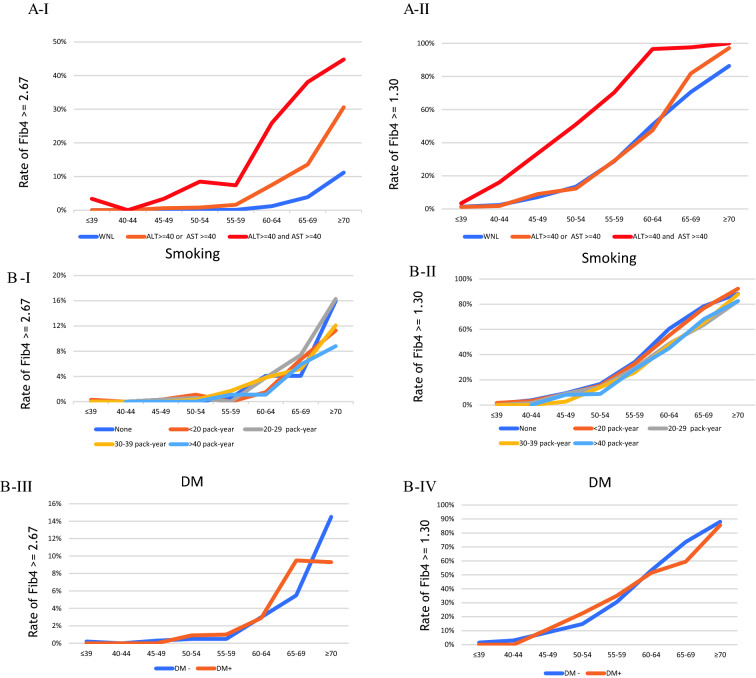

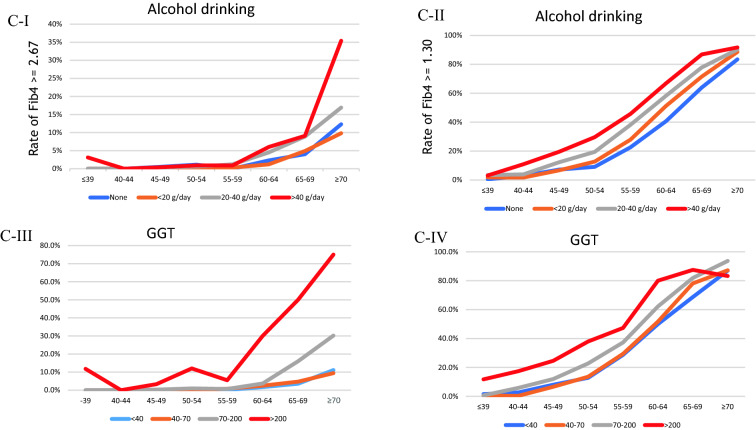

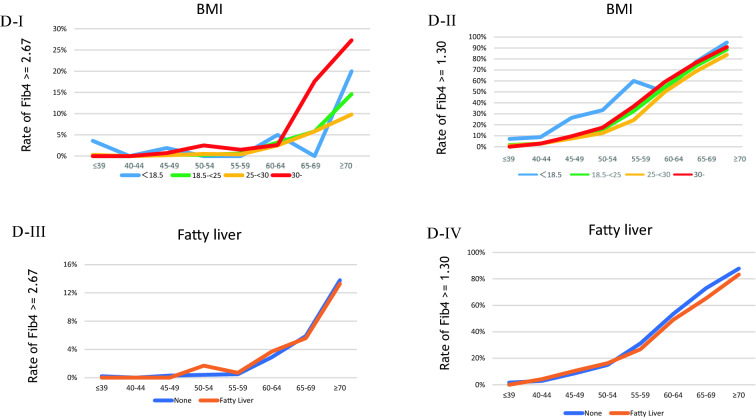

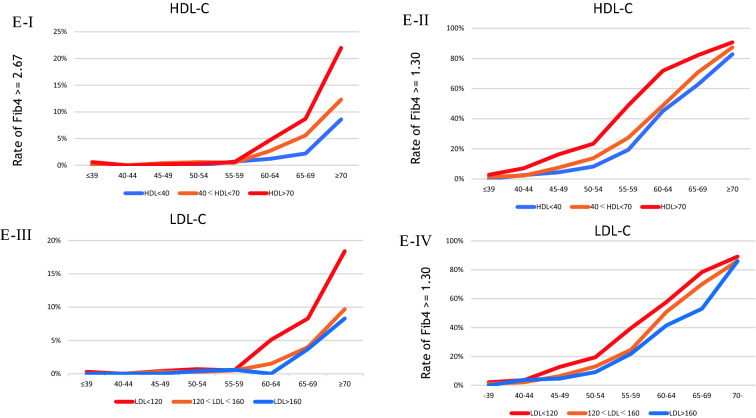
**A** Rate of patients with Fib4 ≥ 2.67 (I) or ≥ 1.3 (II) by each status of ALT and AST abnormalities. Blue, orange, and red line presents ‘with normal limit (WNL),’ ‘ALT ≥ 40 (IU/l) or AST ≥ 40 (IU/l),’ and ‘ALT ≥ 40 (IU/l) and AST ≥ 40 (IU/l),’ respectively. **B** Rate of patients with Fib4 ≥ 2.67 (I) or ≥ 1.3 (II) by each status of smoking and that with Fib4 ≥ 2.67 (III) or ≥ 1.3 (IV) by each current status of DM (diabetes mellitus). In (I) and (II), blue, orange, gray, yellow, or dark blue line presents ‘none of smoking habit,’ ‘ < 20 pack-year,’ ‘20–29 pack-year,’ ‘30–39 pack-year,’ and ≥ ‘40 pack-year,’ respectively. In (III) and (IV), blue or orange line presents ‘no present history of DM’ or ‘present history of DM,’ respectively. **C** Rate of patients with Fib4 ≥ 2.67 (I) or ≥ 1.3 (II) by each status of alcohol drinking and that with Fib4 ≥ 2.67 (III) or ≥ 1.3 (IV) by each status of GGT. In (I) and (II), blue, orange, gray, or red line presents ‘no habit of alcohol drinking,’ ‘ < 20 g (ethanol)/day,’ ‘20 ≤ habit of alcohol drinking < 40,’ or ‘habit of alcohol drinking ≥ 40 g,’ respectively. In III) and IV), blue, orange, gray, or red line presents ‘GGT < 40 (U/l),’ ‘40 ≤ GGT < 70,’ ‘70 ≤ GGT < 200,’ or ‘GGT ≥ 200,’ respectively. **D** Rate of patients with Fib4 ≥ 2.67 (I) or ≥ 1.3 (II) by each status of BMI and that with Fib4 ≥ 2.67 (III) or ≥ 1.3 (IV) by each status of fatty liver. In (I) and (II), blue, green, yellow, or red line presents ‘BMI < 18.5 (kg/m^2^),’ ‘18.5 ≤ BMI < 25,’ ‘25 ≤ BMI < 30,’ or ‘ BMI ≥ 30,’ respectively. In (III) and (IV), blue or orange line presents ‘none of fatty liver’ or ‘present of fatty liver,’ respectively. **E** Rate of patients with Fib4 ≥ 2.67 (I) or ≥ 1.3 (II) by each status of HDL, and that with Fib4 ≥ 2.67 (III) or ≥ 1.3 (IV) by each status of LDL. In (I) and (II), blue, orange or red line presents ‘HDL < 40 (mg/dl),’ ‘40 ≤ HDL < 70,’ or ‘HDL ≥ 70,’ respectively. In (III) and (IV), red, orange or blue line presents ‘LDL < 120 (mg/dl),’ ‘120 ≤ LDL < 160,’ or ‘LDL ≥ 160,’ respectively

In the cross-sectional setting, Fig. 2A (I and II) illustrates the rate of patients with Fib4 ≥ 2.67 and ≥ 1.3 by ALT or AST abnormality. The percentage of Fib4 ≥ 2.67 in patients with both ALT and AST ≥ 40 IU/L per age group was 3%, 0%, 3%, 9%, 7%, 25%, 38%, and 45% in ≤ 39, 40–44, 40–45, 50–54, 55–60, 60–64, 65–69, and ≥ 70 years, respectively. Figure 2B shows the rate of patients with Fib4 ≥ 2.67 and ≥ 1.3 by smoking history (pack years) and diabetes mellitus (DM). The rate was not different in terms of the status of smoking history or present illness of DM. Figure 2C shows the rate of patients with Fib4 ≥ 2.67 or ≥ 1.3 by alcohol drinking and GGT. The Fib4 ≥ 2.67 rate was strictly elevated in heavy drinkers (≥ 40 g/day) aged over 65 years. Meanwhile, the Fib4 ≥ 1.3 rate increased with each daily alcohol intake. Furthermore, the GGT ≥ 200 IU/L rate increased among patients aged > 55 years. Figure 2D shows the rate of patients with Fib4 ≥ 2.67 or ≥ 1.3 by BMI and fatty liver presence. In the age group of > 60 years, the Fib4 ≥ 2.67 rate was higher in those with a BMI ≥ 30 kg/m^2^. However, the rate was not different in terms of the fatty liver status. Figure 2E shows the rate of patients with Fib4 ≥ 2.67 or ≥ 1.3 by HDL and LDL. Interestingly, the rate of FIb4 ≥ 2.67 increased dose dependently in those with high HDL and low LDL.

The OR of the risk for Fib4 ≥ 2.67 was calculated by logistic regression, and Table 1 lists the results. In the table, models 1 and 2 show the alcohol drinking and GGT results. The risk for Fib4 ≥ 2.67 was high in heavy alcohol drinkers (OR = 3.21, 95% CI = 1.38–7.44) but considerably high in patients with GGT ≥ 200 (OR = 29.05, 95% CI = 17.03–49.56). The crude OR of HDL ≥ 70 was 2.32 (95% CI = 1.23–4.39), referred to as HDL < 40. A higher HDL showed an increased risk in the univariate analysis, but the risk was not significant in the multivariate analysis. Regarding LDL, the OR of LDL ≥ 160 was 0.35 (95% CI = 0.16–0.77), referred to as LDL ≥ 120.

**Table 1:** Odds ratio of the risk for FIB4 index ≥ 2.67 calculated by logistic methods

	Crude*				Model 1				Model 2			
Variables	Odds	95% CI		*p*	Odds	95% CI		*p*	Odds	95% CI		*p*
Age					1.19	1.17	1.22	< .001	1.21	1.19	1.24	< .001
Medication												
Hypertension	1.18	0.86	1.61	0.307	1.01	0.72	1.40	0.976	0.86	0.61	1.22	0.390
Diabetes mellitus	0.98	0.60	1.58	0.917	0.92	0.56	1.51	0.738	0.94	0.56	1.57	0.811
Smoking												
Never		Reference				Reference				Reference		
Former	0.89	0.63	1.26	0.503	0.77	0.54	1.11	0.163	0.71	0.49	1.03	0.071
Current	1.37	0.92	2.04	0.121	1.15	0.76	1.73	0.512	0.88	0.57	1.36	0.572
BMI (kg/m^2^)												
< 18.5		Reference				Reference				Reference		
18.5–< 25	0.69	0.30	1.58	0.381	0.92	0.40	2.14	0.844	0.77	0.33	1.84	0.562
25–< 30	0.59	0.25	1.40	0.232	0.89	0.34	2.31	0.805	0.75	0.28	1.99	0.562
30–<	1.73	0.62	4.81	0.292	2.73	0.85	8.78	0.093	2.73	0.83	9.00	0.099
Continuous	1.00	0.96	1.055	0.859	1.06	0.99	1.145	0.105	1.07	1.00	1.153	0.053
Fatty Liver	1.30	0.77	2.20	0.323	1.23	0.66	2.31	0.510	0.85	0.45	1.62	0.628
Abdominal circumstance (cm)												
< 85		Reference				Reference				Reference		
≥ 85	0.96	0.71	1.29	0.724	1.02	0.68	1.53	0.923	0.91	0.60	1.37	0.648
HDL (mg/dl)												
< 40		Reference				Reference				Reference		
40–70	1.27	0.70	2.31	0.435	1.23	0.67	2.28	0.506	1.13	0.60	2.12	0.709
≥ 70	2.32	1.23	4.39	0.009	1.81	0.92	3.58	0.087	1.64	0.82	3.27	0.164
Continuous	1.02	1.01	1.024	0.001	1.01	1.00	1.017	0.173	1.01	1.00	1.017	0.123
LDL (mg/dl)												
< 120		Reference				Reference				Reference		
120–160	0.45	0.33	0.62	0.000	0.52	0.37	0.71	< .001	0.51	0.37	0.72	< .001
≥ 160	0.30	0.14	0.64	0.002	0.36	0.16	0.79	0.011	0.35	0.16	0.77	0.009
Continuous	0.98	0.98	0.985	0.001	0.99	0.98	0.991	0.001	0.98	0.98	0.99	0.001
Alcohol drinking (Ethanol; g/day)												
Never		Reference				Reference						
> 20	2.02	1.45	2.81	0.000	1.83	1.29	2.59	< .001				
20–40	3.04	1.97	4.69	0.000	2.60	1.65	4.09	< .001				
> 40	4.30	1.90	9.72	0.000	3.21	1.38	7.44	0.007				
Continuous	1.53	1.32	1.78	0.000	1.05	0.87	1.258	0.634				
GGT (U/l)												
> 40		Reference								Reference		
40–70	1.17	0.75	1.84	0.494					1.21	0.76	1.93	0.411
70–200	4.02	2.71	5.96	0.000					4.13	2.73	6.26	< .001
≥ 200	30.03	18.07	49.90	0.000					29.05	17.03	49.56	< .001
		*p* for trend < 0.001								p for trend < 0.001		

*BMI* body mass index, *HDL* high-density lipoprotein cholesterol, *LDL* low-density lipoprotein cholesterol, *GGT* gamma-glutamyl transferase

^*^Age-adjusted

**Fig. 3:**
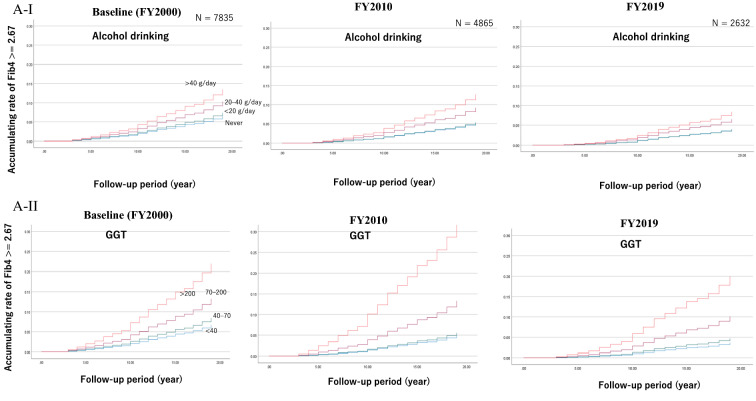

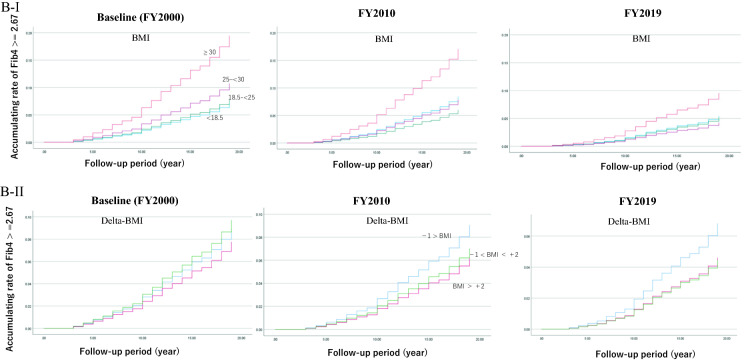

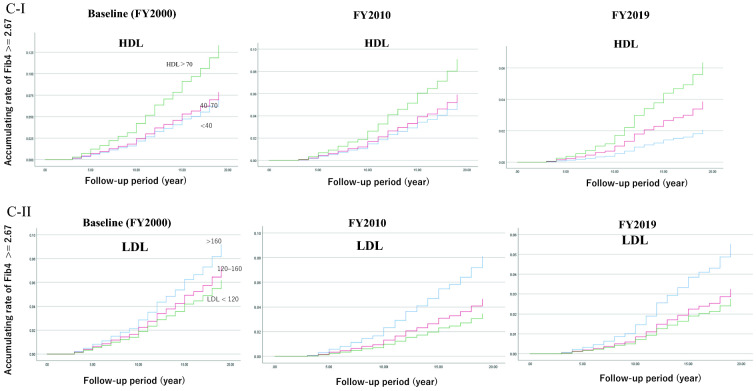
Accumulating rate of Fib4 ≥ 2.67 by Cox analysis. **A** Accumulating rate of Fib4 ≥ 2.67 by each status of alcohol drinking (I) and GGT (II) at baseline (FY2000), FY2010, and FY2019). In (I), blue, green, purple, or orange line presents ‘no habit of alcohol drinking, ‘ < 20 g (ethanol)/day,’ ‘20–39 g,’ or ‘ ≥ 40 g,’ respectively. In (II), blue, green, purple, or orange line presents ‘GGT < 40 (IU/l),’ ‘40 ≤ GGT < 70,’ ‘70 ≤ GGT < 200,’ or ‘GGT ≥ 200,’ respectively. **B** Accumulating rate of Fib4 ≥ 2.67 by each status of BMI (I) and delta-BMI (II) at baseline (FY2000), FY2010, and FY2019). In (I), blue, green, purple, or orange line presents ‘BMI < 18.5 (kg/m^2^),’ ‘18.5 ≤ BMI < 25,’ ‘25 ≤ BMI < 30,’ or ‘ BMI ≥ 30,’ respectively. In (II), blue, green, or purple line presents ‘ delta-BMI < − 1 (kg/m^2^),’ ‘ − 1 ≤ delta-BMI <  + 2,’ or ‘ BMI ≥  + 2,’ respectively. **C** Accumulating rate of Fib4 ≥ 2.67 by each status of HDL (I) and LDL (II) at baseline (FY2000), FY2010, and FY2019). In (I), blue, purple, or green line presents ‘ HDL < 40 (mg/dl),’ ‘ 40 ≤ HDL < 70,’ or ‘ HDL ≥ 70,’ respectively. In (II), green, purple, or blue line presents ‘ LDL < 120 (mg/dl),’ ‘ 120 ≤ LDL < 160,’ or ‘ LDL ≥ 160,’ respectively

Figure 3 shows the results of the retrospective cohort setting obtained by using the COX proportional model. Number of subjects who could be followed up is shown in Additional file 1: Table S1. Figure 3A demonstrates the accumulating rate of Fib4 ≥ 2.67 in terms of alcohol drinking and GGT. The HR was adjusted with age, BMI, HDL, and LDL. Additional file 1: Tables S2 and S3 list the detailed information. The HR of alcohol drinking at 20–40 and ≥ 40 g/day was 1.63 (95% CI = 1.32–2.17) and 2.17 (1.58–2.98), respectively. The HR is also shown in the information obtained at FY2010 and FY2019. The association between alcohol drinking and the Fib4 ≥ 2.67 rate did not change from baseline using the information after 10 and 20 years. However, the relationship of the Fib4 ≥ 2.67 rate with GGT differed. The HRs of those with a high GGT value (≥ 200 IU/L) at baseline, 10 years, and 20 years later were 3.57 (95%CI = 2.36–5.41), 7.65 (95%CI = 5.65–111.12), and 6.04 (95% CI = 3.35–10.91), respectively. Thus, the rate of Fib4 ≥ 2.67 sharply increased among those with a high GGT value (≥ 200) at 10 years after the baseline (Fig. 3A-II).

Figure 3B shows the accumulating rate of Fib4 ≥ 2.67 by the status of BMI and change of BMI (delta-BMI). The HR was adjusted with age, HDL, LDL, and GGT, and detailed information is shown in Additional file 1: Tables S4 and S5. Patients with BMI ≥ 30 kg/m^2^ at baseline or 10 years later had a higher rate of Fib4 ≥ 2.67, but after 20 years, the relationship was no longer observed. In BMI fluctuation, the rate of Fib4 ≥ 2.67 was higher in those who had a BMI decreased by − 1 or less until 10 years after the baseline.

Figure 3C shows the accumulating rate of Fib4 ≥ 2.67 by the status of HDL and LDL. The rate was adjusted with age, BMI, and GGT, and detailed information is shown in Additional file 1: Tables S6 and S7. When HDL > 70 or LDL ≤ 120, the HR of Fib4 ≥ 2.67 was high. In both HDL and LDL cases, the association was seen in a dose-dependent manner. The analysis including or excluding patients with dyslipidemia did not affect the results.

The concordance rate of each category using the kappa value between 4 categories of alcohol drinking habits and 4 categories of GGT at FY2000, FY2010, and FY2019 was 0.081, 0.079, and 0.073, respectively. The concordance rates were 0.476 and 0.421 between alcohol drinking at FY2000 and that at FY2010 and between alcohol drinking at FY2000 and that at FY2019, respectively (Tables 2, 3, 4).

**Table 2:** Cross table of alcohol drinking and GGT at FY2000, FY2010, and FY2019

Alcohol drinking at FY2000	GGT at FY2000 (baseline)					GGT at FY2010					GGT at FY2019			
	> 40 (U/l)	40–70	70–200	≥ 200	Total	> 40	40–70	70–200	≥ 200	Total	> 40	40–70	≥ 200	Total
Never	1088	370	124	11	1593	850	183	66	7	1106	559	103	4	706
	68.3%	23.2%	7.8%	0.7%	100%	76.9%	16.5%	6.0%	0.6%	100%	79%	15%	1%	100%
> 20 g/day	1774	845	504	49	3172	1438	535	298	32	2303	992	325	23	1492
	55.9%	26.6%	15.9%	1.5%	100%	62.4%	23.2%	12.9%	1.4%	100%	67%	22%	2%	100%
20–40 g/day	751	643	574	83	2051	653	425	350	64	1492	479	261	36	973
	36.6%	31.4%	28.0%	4.0%	100%	43.8%	28.5%	23.5%	4.3%	100%	49%	27%	4%	100%
> 40 g/day	240	311	386	66	1003	227	215	227	44	713	181	117	31	460
	23.9%	31.0%	38.5%	6.6%	100%	31.8%	30.2%	31.8%	6.2%	100%	39.3%	25.4%	6.7%	100%
Total	3853	2169	1588	209	7819	3168	1358	941	147	5614	2211	806	94	3631
	49.3%	27.7%	20.3%	2.7%	100%	56.4%	24.2%	16.8%	2.6%	100%	60.9%	22.2%	2.6%	100%
Kappa					0.081					0.079				0.073
Spearman's coefficient					0.324					0.316				0.289

**Table 3:** Cross table of alcohol drinking at baseline, at FY2010, and FY2019

Alcohol drinking at FY2000	Alcohol drinking at FY2010					Alcohol drinking at FY2019				
	Never	> 20 g/day	20–40 g/day	> 40 g/day	Total	Never	> 20 g/day	20–40 g/day	> 40 g/day	Total
Never	910	164	20	12	1106	553	120	26	7	706
	82.3%	14.8%	1.8%	1.1%	100%	78.3%	17.0%	3.7%	1.0%	100%
> 20 g/day	355	1524	364	60	2303	277	896	263	56	1492
	15.4%	66.2%	15.8%	2.6%	100%	18.6%	60.1%	17.6%	3.8%	100%
20–40 g/day	49	479	749	215	1492	57	299	477	140	973
	3.3%	32.1%	50.2%	14.4%	100%	5.9%	30.7%	49.0%	14.4%	100%
> 40 g/day	17	100	261	335	713	15	55	183	207	460
	2.4%	14.0%	36.6%	47.0%	100%	3.3%	12.0%	39.8%	45.0%	100%
Total	1331	2267	1394	622	5614	902	1370	949	410	3631
	23.7%	40.4%	24.8%	11.1%	100%	24.8%	37.7%	26.1%	11.3%	100%
Kappa					0.476					0.421
Spearman's coefficient					0.706					0.659

**Table 4:** Cross table of GGT at baseline, at FY2010, and FY2019

GGT at FY2000 (baseline)	GGT at FY2010					GGT at FY2019				
	> 40 (U/l)	40–70	70–200	≥ 200	Total	> 40	40–70	70–200	≥ 200	Total
> 40 (U/l)	2354	344	92	1	2791	1505	236	67	3	1811
	84.3%	12.3%	3.3%	0.0%	100%	83.1%	13.0%	3.7%	0.2%	100%
40–70	642	633	256	15	1546	500	333	163	21	1017
	41.5%	40.9%	16.6%	1.0%	100%	49.2%	32.7%	16.0%	2.1%	100%
70–200	175	374	530	80	1159	202	227	249	48	726
	15.1%	32.3%	45.7%	6.9%	100%	27.8%	31.3%	34.3%	6.6%	100%
≥ 200	5	11	66	51	133	9	14	41	22	86
	3.8%	8.3%	49.6%	38.3%	100%	10.5%	16.3%	47.7%	25.6%	100%
Total	3176	1362	944	147	5629	2216	810	520	94	3640
	56.4%	24.2%	16.8%	2.6%	100%	60.9%	22.3%	14.3%	2.6%	100%
Kappa					0.630					0.306
Spearman's coefficient					0.408					0.515

## Discussion

This study demonstrated that Fib4 could be an effect marker in identifying patients who need assessment for ALD by using GGT at the same time as an exposure and effect marker for estimating alcohol consumption. In ALD, the AST/ALT ratio increases and platelets decrease as liver fibrosis progresses [8]; thus, theoretically, Fib4 could be an effect marker. In this study, when GGT exceeded 200 IU/L in a group of patients after 10 years, the rate of Fib4 ≥ 2.67 considerably elevated. Currently, it is unlikely that an examinee will consult a hepatologist solely due to a high GGT on health examinations. If future clinical studies reveal alcoholic parenchymal damage in the livers of patients with elevated levels of both Fib4 and GGT, a follow-up program for subjects with high GGT values would be developed.

In this study, although high BMI (≥ 30 kg/m^2^) indicated a risk for Fib4 elevation, our results showed a difference between such risk and the status of fatty liver, and Fib4 values were higher in those who lost weight than in those who gained weight. According to our results and recent findings [7], Fib4 may be difficult to interpret for NAFLD in the general population. NAFLD is defined as the consumption of ≤ 30 g of alcohol per day [22], but in the case of alcoholic liver injury, ≥ 60 g is consumed [22]. During our study period, numerous male workers consumed alcohol at 30–60 g/day, and their health management is also important. Fib4 seems to have a significance as a marker for liver fibrosis because of the addition of platelet levels. Liver function tests, including ALT, AST, and GGT, are strongly associated with fatty liver in conjunction with metabolic syndrome [24] or ALD [8]. Considering that the prevalence of viral hepatitis has reduced [25], the interest has now shifted to NASH [22]. In addition, ALD is categorized as addiction and is treated by a special psychiatric field. From these points, ALD at a mild stage is definitely overlooked [8].

GGT increases not only by alcohol consumption but also by metabolic syndrome and enzyme-inducing drugs [13]. Baseline GGT level is positively and strongly associated with the risk for metabolic syndrome in a nonlinear dose–response manner [26, 27]. Several epidemiologic studies have also demonstrated important advances in the definition of the associations between serum GGT level and the risk of overall mortality, coronary heart disease, type 2 DM, stroke, and chronic kidney disease [12, 28–30]. The regulatory mechanism of GGT expression has been already been widely investigated. In addition, the 5′‐untranslated regions of mRNAs of the enzyme differ in a tissue‐specific manner but share a common protein-coding region, and the tissue‐specific and developmental stage-specific expression, as well as hepatic induction, is conferred by different promoters [31]. By light microscopy, alcoholic liver samples had a marked GGT activity in the bile canaliculi and a diffuse activity in the cytoplasm [32]. Recently, the GGT/albumin ratio included gamma‐glutamyl transpeptidase, and albumin is a novel inflammatory marker [33]. These findings on GGT are mainly involved in glutathione metabolism and cellular protection against oxidative damage [13].

This study also found interesting results in lipid metabolism. For instance, Fib4 is associated with dyslipidemia. Thus, we excluded patients with medication for dyslipidemia. Low HDL-C has been associated with NAFLD and end-stage hepatitis [34, 35]. Recently, the total cholesterol/HDL-C ratio was reported to be a predictive marker of NAFLD [36], and the triglyceride/HDL-C ratio as a predictive marker of metabolic-associated fatty liver disease (MAFLD) [37]. While the current study found that Fib4 increment positively correlated with HDL-C, a previous study reported an inverse relationship between Fib4 and GGT/HDL ratio, which increases with MAFLD [38]. One possible reason for these phenomena is the inadequate model-based statistical adjustment for alcohol consumption, which can elevate HDL-C; HDL-C is known to be an objective maker for alcohol consumption, independent of self-report [39, 40]. According to our findings, Fib4 might be associated with the risk of liver disease by alcoholic consumption.

In ALD, alcoholic hepatitis progresses to alcoholic steatohepatitis, leading to cirrhosis in some patients. The vast majority (90–100%) of chronic heavy drinkers develop alcoholic fatty liver disease. However, only 10–20% develop advanced ALD, and individual differences in its susceptibility for ALD are still poorly understood [8]. Although GGT levels are associated with alcohol consumption, the reported levels only correlate moderately with alcohol consumption (*r* = 0.30–0.40 in males, 0.15–0.30 in females); additionally, GGT level elevation is different between individuals with the same amount of alcohol consumed [41]. In the present study, the kappa value between the self-report of alcohol drinking and the serum level of GGT was very low (0.081). However, as mentioned above, GGT elevation indicates a risk for liver-related mortality [29]. Thus, GGT (GGT responder) increase may be a good marker of individual susceptibility for liver damage by oxidative stress, including the alcohol metabolism in ALD. However, only GGT abnormalities are found in health examinations, and any follow-up measures have not been established. Clinically clarifying the relationship between Fib4, GGT, and ALD would increase the significance of GGT measurement in health examinations.

Patients with ALD have been treated mainly by addiction specialists in the psychiatric field as alcohol dependence. Recently, harm reduction by reducing alcohol consumption, which has been used as a treatment approach in Europe [42], has gained recognition in Japan [43]. Treatment with anti-alcohol drugs, such as nalmefene, has also advanced [44], and it can be prescribed not only by specialized psychiatrists but also by hepatologists. Therefore, when ALD is suspected from the health examination results, patients must be actively recommended to seek consultation to hepatologists. In this study, analysis was performed using threshold values of 2.67 for Fib4 [23] and 200 IU/L for GGT; nevertheless, it will be necessary in the future to examine these cutoff values among general workers.

Regarding the strength of this study, it was verified by a cross-sectional study and a 20-year longitudinal study. However, given that the limitation is a follow-up survey in the workplace, selection bias is possible because a healthy-worker effect cannot be denied. In addition, although GGT ≥ 200 IU/L and Fib4 ≥ 2.67 are proposed, clinical studies are required in this respect. Additionally, clinically elastorgraphy verification, histopathological examinations, and investigation on long-term outcomes should be conducted.

In conclusion, Fib4 combined with GGT could be a useful effect marker for alcoholic liver injury. Although the examinee does not often refer to a hepatologist merely because GGT is high in health examinations, liver parenchymal injury might be considered if both Fib4 and GGT increase. On the basis of our result, we proposed that alcoholic liver injury occurs if the GGT value exceeds 200 IU/L and Fib4 is 2.67 or more. Thus, Fib4 could be an effect marker on alcoholic liver injury, together with GGT in health examinations. However, further clinical evaluation studies are required.

## Supplementary Information

**Additional file 1: Table S1.** Number of follow-up. **Table S2.** Hazard ratio calculated by COX model for Fib4 index ≥ 2.67 using information on alcohol drinking habits at baseline, FY2010, and Fy2019. **Table S3.** Hazard ratio calculated by COX model for Fib4 index ≥ 2.67 using information on GGT at baseline, FY2010, and Fy2019. **Table S4.** Hazard ratio calculated by COX model for Fib4 index ≥ 2.67 using information on BMI at baseline, FY2010, and Fy2019. **Table S5.** Hazard ratio calculated by COX model for Fib4 index ≥ 2.67 using information on change of BMI at baseline, FY2010, and Fy2019. **Table S6.** Hazard ratio calculated by COX model for Fib4 index ≥ 2.67 using information on HDL at baseline, FY2010, and Fy2019. **Table S7.** Hazard ratio calculated by COX model for Fib4 index ≥ 2.67 using information on HDL at baseline, FY2010, and Fy2019.

## Data Availability

The data sets during and/or analyzed during the current study available from the corresponding author on reasonable request.

## Abbreviations

PI: Present illness
PH: Past history
FY: Fiscal year
Fib4: Fib4 index
AST: Aspartate aminotransferase
ALT: Alanine aminotransferase
NAFLD: Nonalcoholic fatty liver disease
NAFL: Nonalcoholic fatty liver
NASH: Nonalcoholic steatohepatitis
HCC: Hepatocellular carcinoma
ALD: Alcoholic liver disease
HDL: High-density lipoprotein cholesterol
LDL: Low-density lipoprotein cholesterol
GGT: Gamma-glutamyl transferase
OR: Odds ratio
ORs: Odds ratios
CI: 95% Confidence interval
BMI: Body mass index
HR: Hazard ratio
DM: Diabetes mellitus
MAFLD: Metabolic-associated fatty liver disease

## References

[1] Sterling RK, Lissen E, Clumeck N, Sola R, Correa MC, Montaner J, Mark SS, Torriani FJ, Dieterich DT, Thomas DL (2006). Development of a simple noninvasive index to predict significant fibrosis in patients with HIV/HCV coinfection. Hepatology.

[2] Targher G, Tilg H, Byrne CD (2021). Non-alcoholic fatty liver disease: a multisystem disease requiring a multidisciplinary and holistic approach. Lancet Gastroenterol Hepatol.

[3] Balakrishnan M, Loomba R (2020). The role of noninvasive tests for differentiating NASH From NAFL and diagnosing advanced fibrosis among patients with NAFLD. J Clin Gastroenterol.

[4] Kechagias S, Ekstedt M, Simonsson C, Nasr P. Non-invasive diagnosis and staging of non-alcoholic fatty liver disease. Hormones (Athens). 2022. 10.1007/s42000-022-00377-8 PMC9464753 35661987

[5] Xiao G, Zhu S, Xiao X, Yan L, Yang J, Wu G (2017). Comparison of laboratory tests, ultrasound, or magnetic resonance elastography to detect fibrosis in patients with nonalcoholic fatty liver disease: a meta-analysis. Hepatology.

[6] Naghavi M, Abajobir AA, Abbafati C, Abbas KM, Abd-Allah F, Abera SF, Aboyans V, Adetokunboh O, Afshin A, Agrawal A (2017). Global, regional, and national age-sex specific mortality for 264 causes of death, 1980–2016: a systematic analysis for the Global Burden of Disease Study 2016. The Lancet.

[7] Sugiyama A, Kurisu A, E B, Ouoba S, Ko K, Rakhimov A, Akita T, Harakawa T, Sako T, Koshiyama M et al. Distribution of FIB-4 index in the general population: analysis of 75,666 residents who underwent health checkups. BMC Gastroenterol. 2022, 22(1):241. 10.1186/s12876-022-02290-1 PMC9101936 35562658

[8] Seitz HK, Bataller R, Cortez-Pinto H, Gao B, Gual A, Lackner C, Mathurin P, Mueller S, Szabo G, Tsukamoto H (2018). Alcoholic liver disease. Nat Rev Dis Primers.

[9] Kamper-Jørgensen M, Grønbaek M, Tolstrup J, Becker U (2004). Alcohol and cirrhosis: dose–response or threshold effect?. J Hepatol.

[10] Corrao G, Bagnardi V, Zambon A, La Vecchia C (2004). A meta-analysis of alcohol consumption and the risk of 15 diseases. Prev Med.

[11] Popham RE, Schmidt W (1981). Words and deeds: the validity of self-report data on alcohol consumption. J Stud Alcohol.

[12] Watson CG, Tilleskjor C, Hoodecheck-Schow EA, Pucel J, Jacobs L (1984). Do alcoholics give valid self-reports?. J Stud Alcohol.

[13] Whitfield JB (2001). Gamma glutamyl transferase. Crit Rev Clin Lab Sci.

[14] Scouller K, Conigrave KM, Macaskill P, Irwig L, Whitfield JB (2000). Should we use carbohydrate-deficient transferrin instead of gamma-glutamyltransferase for detecting problem drinkers? A systematic review and metaanalysis. Clin Chem.

[15] Sakaguchi S, Takahashi S, Sasaki T, Kumagai T, Nagata K (2011). Progression of alcoholic and non-alcoholic steatohepatitis: common metabolic aspects of innate immune system and oxidative stress. Drug Metab Pharmacokinet.

[16] Fukai K, Terauchi R, Noro T, Ogawa S, Watanabe T, Nakagawa T, Honda T, Watanabe Y, Furuya Y, Hayashi T (2022). Real-time risk score for glaucoma mass screening by spectral domain optical coherence tomography: development and validation. Transl Vis Sci Technol.

[17] Honda T, Nakagawa T, Watanabe Y, Hayashi T, Nakano T, Horie S, Tatemichi M (2019). Association between information and communication technology use and ocular axial length elongation among middle-aged male workers. Sci Rep.

[18] Nakano T, Hayashi T, Nakagawa T, Honda T, Owada S, Endo H, Tatemichi M (2017). Applicability of automatic spectral domain optical coherence tomography for glaucoma mass screening. Clin Ophthalmol.

[19] Nakano T, Hayashi T, Nakagawa T, Honda T, Owada S, Endo H, Tatemichi M (2018). Increased incidence of visual field abnormalities as determined by frequency doubling technology perimetry in high computer users among Japanese workers: a retrospective cohort study. J Epidemiol.

[20] Watanabe Y, Nakagawa T, Fukai K, Honda T, Furuya H, Hayashi T, Tatemichi M (2022). Descriptive study of chest x-ray examination in mandatory annual health examinations at the workplace in Japan. PLoS ONE.

[21] Fukai K, Kuwahara K, Chen S, Eguchi M, Kochi T, Kabe I, Mizoue T (2020). The association of leisure-time physical activity and walking during commuting to work with depressive symptoms among Japanese workers: a cross-sectional study. J Occup Health.

[22] Watanabe S, Hashimoto E, Ikejima K, Uto H, Ono M, Sumida Y, Seike M, Takei Y, Takehara T, Tokushige K (2015). Evidence-based clinical practice guidelines for nonalcoholic fatty liver disease/nonalcoholic steatohepatitis. J Gastroenterol.

[23] Shah AG, Lydecker A, Murray K, Tetri BN, Contos MJ, Sanyal AJ (2009). Comparison of noninvasive markers of fibrosis in patients with nonalcoholic fatty liver disease. Clin Gastroenterol Hepatol.

[24] Eslam M, Newsome PN, Sarin SK, Anstee QM, Targher G, Romero-Gomez M, Zelber-Sagi S, Wai-Sun Wong V, Dufour JF, Schattenberg JM (2020). A new definition for metabolic dysfunction-associated fatty liver disease: an international expert consensus statement. J Hepatol.

[25] Tanaka J, Kurisu A, Ohara M, Ouoba S, Ohisa M, Sugiyama A, Wang ML, Hiebert L, Kanto T, Akita T (2022). Burden of chronic hepatitis B and C infections in 2015 and future trends in Japan: a simulation study. Lancet Reg Health West Pac.

[26] Ma Q, Liao X, Shao C, Lin Y, Wu T, Sun Y, Feng ST, Ye J, Zhong B (2021). Normalization of γ-glutamyl transferase levels is associated with better metabolic control in individuals with nonalcoholic fatty liver disease. BMC Gastroenterol.

[27] Kunutsor SK, Apekey TA, Seddoh D (2015). Gamma glutamyltransferase and metabolic syndrome risk: a systematic review and dose-response meta-analysis. Int J Clin Pract.

[28] Kim YG, Han K, Jeong JH, Roh SY, Choi YY, Min K, Shim J, Choi JI, Kim YH. Metabolic syndrome, gamma-glutamyl transferase, and risk of sudden cardiac death. J Clin Med. 2022; 11(7). 10.3390/jcm11071781 PMC8999874 35407389

[29] Ho FK, Ferguson LD, Celis-Morales CA, Gray SR, Forrest E, Alazawi W, Gill JM, Katikireddi SV, Cleland JG, Welsh P (2022). Association of gamma-glutamyltransferase levels with total mortality, liver-related and cardiovascular outcomes: a prospective cohort study in the UK Biobank. EClinicalMedicine.

[30] Li S, Wang A, Tian X, Zuo Y, Meng X, Zhang Y. Elevated gamma-glutamyl transferase levels are associated with stroke recurrence after acute ischemic stroke or transient ischemic attack. CNS Neurosci Ther. 2022. 10.1111/cns.13909 PMC9437228 35789538

[31] Ikeda Y, Taniguchi N (2005). Gene expression of gamma-glutamyltranspeptidase. Methods Enzymol.

[32] Ishii H, Ebihara Y, Okuno F, Munakata Y, Takagi T, Arai M, Shigeta S, Tsuchiya M (1986). Gamma-Glutamyl transpeptidase activity in liver of alcoholics and its localization. Alcohol Clin Exp Res.

[33] Li H, Liu R, Li J, Li J, Wu H, Wang G, Li Z, Li D (2022). Prognostic significance of gamma-glutamyl transpeptidase to albumin ratio in patients with intrahepatic cholangiocarcinoma after hepatectomy. J Cell Mol Med.

[34] Deprince A, Haas JT, Staels B (2020). Dysregulated lipid metabolism links NAFLD to cardiovascular disease. Mol Metab.

[35] Wang Y, Shen W, Huang F, Yu C, Xi L, Gao J, Yin M, Liu X, Lin J, Liu L (2022). HDL-C levels added to the MELD score improves 30-day mortality prediction in Asian patients with cirrhosis. J Int Med Res.

[36] Ren XY, Shi D, Ding J, Cheng ZY, Li HY, Li JS, Pu HQ, Yang AM, He CL, Zhang JP (2019). Total cholesterol to high-density lipoprotein cholesterol ratio is a significant predictor of nonalcoholic fatty liver: Jinchang cohort study. Lipids Health Dis.

[37] Liu Z, He H, Dai Y, Yang L, Liao S, An Z, Li S (2022). Comparison of the diagnostic value between triglyceride-glucose index and triglyceride to high-density lipoprotein cholesterol ratio in metabolic-associated fatty liver disease patients: a retrospective cross-sectional study. Lipids Health Dis.

[38] Xing Y, Chen J, Liu J, Ma H (2022). Associations between GGT/HDL and MAFLD: a cross-sectional study. Diabetes Metab Syndr Obes.

[39] Berger D, Williams EC, Bryson CL, Rubinsky AD, Bradley KA (2013). Alcohol questionnaires and HDL: screening scores as scaled markers of alcohol consumption. Alcohol.

[40] Høiseth G, Hilberg T, Trydal T, Husa A, Vindenes V, Bogstrand ST (2022). The alcohol marker phosphatidylethanol is closely related to AST, GGT, ferritin and HDL-C. Basic Clin Pharmacol Toxicol.

[41] Sillanaukee P, Massot N, Jousilahti P, Vartiainen E, Sundvall J, Olsson U, Poikolainen K, Pönniö M, Allen JP, Alho H (2000). Dose response of laboratory markers to alcohol consumption in a general population. Am J Epidemiol.

[42] Kiefer F, Batra A, Petersen KU, Ardern IS, Tananska D, Bischof G, Funke W, Lindenmeyer J, Mueller S, Preuss UW (2022). German guidelines on screening, diagnosis, and treatment of alcohol use disorders: update 2021. Eur Addict Res.

[43] New guidelines for the diagnosis and treatment of alcohol and drug use disorders Shinko Igaku Shuppansha.; 2018.

[44] Miyata H, Takahashi M, Murai Y, Tsuneyoshi K, Hayashi T, Meulien D, Sørensen P, Higuchi S (2019). Nalmefene in alcohol-dependent patients with a high drinking risk: randomized controlled trial. Psychiatry Clin Neurosci.

