# Supplementary material for: Significance of Fib4 index as an indicator of alcoholic hepatotoxicity in health examinations among Japanese male workers: a cross-sectional and retrospectively longitudinal study
Source: Eur J Med Res. 2023 Jan 18;28:31. doi: 10.1186/s40001-022-00976-6 (PMC9847145; doi:10.1186/s40001-022-00976-6)
Supplement: Supplementary file 1 [file 40001_2022_976_MOESM1_ESM.docx]

Table S1. Number of follow-up

Follow-up year	3	4	5	6	7	8	9	10	11	12	13	14	15	16	17	18	19	Total
Number	671	489	402	337	323	283	477	256	366	339	257	201	234	172	230	176	2632	7845
Fib4 ≥ 2.67	32	31	36	28	42	25	24	50	34	36	21	22	23	10	19	21	20	507

Table S2. Hazard ratio calculated by COX model for Fib4-index ≥2.67 using information on alcohol drinking habits at baseline, FY2010, and FY2019

			Information on Baseline				Information on FY2010				Information on FY2019			
			n = 7835				n = 4865				n = 2632			
			HR	95% CI		*p*	HR	95% CI		*p*	HR	95% CI		*p*
Alcohol drinking	Never	Model 1	reference				reference				reference			
(Ethanol; g/day)	>20 g/day		1.19	0.90	1.58	0.225	1.09	0.81	1.46	0.583	1.03	0.70	1.51	0.901
	20–40 g/day		1.80	1.36	2.38	<.001	2.02	1.51	2.69	<.001	1.86	1.28	2.70	0.001
	>40 g/day		2.50	1.84	3.39	<.001	2.84	2.05	3.95	<.001	2.41	1.54	3.78	<.001
Alcohol drinking	Never	Model 2	reference				reference				reference			
(Ethanol; g/day)	>20 g/day		1.11	0.83	1.47	0.481	1.02	0.76	1.38	0.884	0.94	0.64	1.39	0.747
	20-40 g/day		1.50	1.12	1.99	0.006	1.74	1.29	2.35	<.001	1.56	1.06	2.30	0.024
	>40 g/day		1.92	1.40	2.64	<.001	2.39	1.70	3.36	<.001	1.98	1.25	3.16	0.004

Model1: Adjusted with age and BMI

Model2:Adjusted with age, BMI, HDL, and LDL

Table S3. Hazard ratio calculated by COX model for Fib4-index ≥2.67 using information on GGT at baseline, FY2010, and FY2019

			Information on Baseline				Information on FY2010				Information on FY2019			
			n = 7835				n = 4865				n = 2632			
			HR	95% CI		*p*	HR	95% CI		*p*	HR	95% CI		*p*
GGT (U/l)	>40	Model 1	reference				reference				reference			
	40–70		1.26	1.00	1.60	0.049	1.14	0.86	1.51	0.358	1.30	0.90	1.87	0.158
	70–200		2.15	1.71	2.70	<.001	2.86	2.24	3.66	<.001	2.92	2.08	4.10	<.001
	≥200		3.90	2.66	5.72	<.001	9.18	6.49	12.99	<.001	6.68	3.92	11.38	<.001
GGT (U/l)	>40	Model 2	reference				reference				reference			
	40–70		1.24	0.98	1.56	0.078	1.12	0.84	1.49	0.432	1.30	0.90	1.87	0.158
	70–200		1.96	1.56	2.47	<.001	2.77	2.16	3.57	<.001	2.81	1.98	3.97	<.001
	≥200		3.14	2.07	4.76	<.001	7.41	5.08	10.79	<.001	5.66	3.13	10.26	<.001

Model1: Adjusted with age, BMI,

Model2: Adjusted with age, BMI, HDL, and LDL

Table S4. Hazard ratio calculated by COX model for Fib4-index ≥2.67 using information on BMI at baseline, FY2010, and FY2019

			Information on Baseline				Information on FY2010				Information on FY2019			
			n = 7835				n = 4865				n = 2632			
			HR	95% CI		*p*	HR	95% CI		*p*	HR	95% CI		*p*
BMI (kg/m2)	<18.5	Model 1	reference				reference				reference			
	18.5–25		0.79	0.48	1.28	0.339	0.49	0.29	0.85	0.010	0.81	0.36	1.85	0.622
	25–30		0.91	0.54	1.51	0.702	0.57	0.32	0.99	0.045	0.64	0.27	1.48	0.294
	≥30		1.52	0.72	3.18	0.269	1.17	0.58	2.35	0.661	1.24	0.48	3.24	0.655
BMI (kg/m2)	<18.5	Model 2	reference				reference				reference			
	18.5–25		1.10	0.67	1.80	0.719	0.70	0.39	1.24	0.216	0.94	0.41	2.13	0.873
	25–30		1.54	0.90	2.63	0.112	0.92	0.51	1.69	0.797	0.77	0.33	1.83	0.560
	≥30		2.94	1.37	6.29	0.006	2.12	1.01	4.44	0.047	1.80	0.68	4.77	0.239

Model1: Adjusted with age and GGT

Model2: Adjusted with age, GGT, HDL, and LDL

Table S5. Hazard ratio of COX model for Fib4-index ≥2.67 using information on change of BMI at baseline, FY2010, and FY2019

			Information on Baseline *				Information on FY2010**				Information on FY2019***			
			n = 7835				n = 4865				n = 2632			
			HR	95% CI		*p*	HR	95% CI		*p*	HR	95% CI		*p*
Change of BMI	<−1	Model 1	reference				reference				reference			
(kg/m2)	−1 to＋2		0.88	0.67	1.15	0.333	0.64	0.50	0.82	<.001	0.68	0.49	0.95	0.022
	＞+2		1.12	0.73	1.71	0.616	0.76	0.54	1.09	0.138	0.71	0.48	1.05	0.082
Change of BMI	<－1	Model 2	reference				reference				reference			
(kg/m2)	－1 to＋2		0.81	0.62	1.06	0.124	0.63	0.48	0.82	<.001	0.64	0.46	0.89	0.008
	＞+2		1.01	0.65	1.55	0.982	0.70	0.49	1.02	0.060	0.61	0.41	0.92	0.017

Model1: Adjusted with age and GGT

Model2: Adjusted with age, GGT, HDL, and LDL

* change of BMI = BMI at 2005 − BMI at 2000

** change of BMI = BMI at 2010 － BMI at 2000

*** change of BMI = BMI at 2019 － BMI at 2000

Table S6. Hazard ratio calculated by COX model for Fib4-index ≥2.67 using information on HDL at baseline, FY2010, and FY2019

			Information on Baseline				Information on FY2010				Information on FY2019			
			n = 6567				n = 3908				n = 2908			
			HR	95% CI		*p*	HR	95% CI		*p*	HR	95% CI		*p*
HDL (mg/dl)	<40	Model 1	reference				reference				reference			
	40–70		1.19	0.87	1.63	0.272	1.23	0.83	1.83	0.303	2.19	0.89	5.38	0.089
	≥70		2.17	1.50	3.16	<.001	2.07	1.32	3.27	0.002	3.68	1.44	9.41	0.007
HDL (mg/dl)	<40	Model 2	reference				reference				reference			
	40–70		1.19	0.86	1.64	0.298	1.20	0.80	1.80	0.373	1.93	0.79	4.75	0.152
	≥70		1.95	1.33	2.86	<.001	1.95	1.23	3.10	0.005	3.22	1.26	8.19	0.014

Model1: Adjusted with age, BMI, and GGT

Model2: Adjusted with age, BMI, GGT, and LDL

Table S7. Hazard ratio calculated by COX model for Fib4-index ≥2.67 using information on HDL at baseline, FY2010, and FY2019

			Information on Baseline				Information on FY2010				Information on FY2019			
			n = 6567				n = 3908				n = 2908			
			HR	95% CI		*p*	HR	95% CI		*p*	HR	95% CI		*p*
LDL (mg/dl)	<120	Model 1	reference				reference				reference			
	120–160		0.71	0.57	0.88	0.002	0.53	0.41	0.67	<.001	0.54	0.38	0.76	<.001
	≥160		0.62	0.35	1.09	0.096	0.36	0.19	0.68	0.002	0.46	0.21	0.98	0.045
LDL (mg/dl)	<120	Model 2	reference				reference				reference			
	120–160		0.74	0.59	0.92	0.008	0.55	0.43	0.70	<.001	0.58	0.41	0.82	0.002
	≥160		0.67	0.38	1.17	0.157	0.39	0.20	0.73	0.004	0.49	0.23	1.07	0.072

Model1: Adjusted with age, BMI, and GGT

Model2: Adjusted with age, BMI, GGT, and LDL
